# A Convenient Synthetic Method to Improve Immunogenicity of *Mycobacterium tuberculosis* Related T-Cell Epitope Peptides

**DOI:** 10.3390/vaccines7030101

**Published:** 2019-08-27

**Authors:** Kata Horváti, Bernadett Pályi, Judit Henczkó, Gyula Balka, Eleonóra Szabó, Viktor Farkas, Beáta Biri-Kovács, Bálint Szeder, Kinga Fodor

**Affiliations:** 1 MTA-ELTE Research Group of Peptide Chemistry, Eötvös Loránd University, Hungarian Academy of Sciences, Budapest 1117, Hungary; 2 Institute of Chemistry, Eötvös Loránd University, Budapest 1117, Hungary; 3 National Biosafety Laboratory, National Public Health Center, Budapest 1097, Hungary; 4 Department of Pathology, University of Veterinary Medicine, Budapest 1078, Hungary; 5 Laboratory of Bacteriology, Korányi National Institute for Tuberculosis and Respiratory Medicine, Budapest 1122, Hungary; 6 MTA-ELTE Protein Modelling Research Group, Eötvös Loránd University, Hungarian Academy of Sciences, Budapest 1117, Hungary; 7 Research Centre for Natural Sciences, Hungarian Academy of Sciences, Budapest 1117, Hungary; 8 Department of Laboratory Animal and Animal Protection, University of Veterinary Medicine, Budapest 1078, Hungary

**Keywords:** T-cell epitope, maleimide conjugation, *Mycobacterium tuberculosis*, peptide-based vaccines, tuftsin

## Abstract

Epitopes from different proteins expressed by *Mycobacterium tuberculosis* (*Rv1886c*, *Rv0341*, *Rv3873*) were selected based on previously reported antigenic properties. Relatively short linear T-cell epitope peptides generally have unordered structure, limited immunogenicity, and low *in vivo* stability. Therefore, they rely on proper formulation and on the addition of adjuvants. Here we report a convenient synthetic route to induce a more potent immune response by the formation of a trivalent conjugate in spatial arrangement. Chemical and structural characterization of the vaccine conjugates was followed by the study of cellular uptake and localization. Immune response was assayed by the measurement of splenocyte proliferation and cytokine production, while vaccine efficacy was studied in a murine model of tuberculosis. The conjugate showed higher tendency to fold and increased internalization rate into professional antigen presenting cells compared to free epitopes. Cellular uptake was further improved by the incorporation of a palmitoyl group to the conjugate and the resulted **pal-A(P)I** derivative possessed an internalization rate 10 times higher than the free epitope peptides. Vaccination of CB6F1 mice with free peptides resulted in low T-cell response. In contrast, significantly higher T-cell proliferation with prominent expression of IFN-γ, IL-2, and IL-10 cytokines was measured for the palmitoylated conjugate. Furthermore, the **pal-A(P)I** conjugate showed relevant vaccine efficacy against *Mycobacterium tuberculosis* infection.

## 1. Introduction

Vaccination with whole pathogens elicits long-lasting immunity and robust immune response. However, immunization with subunit vaccines using purified and specific antigens became an attractive alternative because of manufacturing, safety, and stability considerations. Efficient vaccination is still challenging in a variety of infectious diseases where the causative agent shows high antigenic diversity due to fast mutation rate or metamorphosis. Subunit vaccines that can combine multiple antigens derived from different stages of a pathogen’s life cycle hold promise for overcoming the major obstacles. 

The premise of the epitope-based vaccine approach is the more focused and more functional protective immunity [1]. Epitopes are minimal antigenic determinants of proteins. They have the capacity to activate B- and T-cells and consequently elicit humoral and/or cellular immune response against the target pathogen. Synthetic peptides have great potential for pharmaceutical application, as they are compounds with high selectivity and specificity, good tolerability, well-defined chemical composition, stability, solubility, and affordable large-scale production [2]. However, we need to deal with disadvantages such as the fast in-vivo degradation, low systemic stability, lack of tissue-specific penetration, loss of native conformation, and low immunogenicity. Despite these generic disadvantages, there are approximately 140 peptide therapeutics being evaluated in clinical trials [3]. Chemical modifications such as cyclisation, non-native amino acid incorporation, conjugation to carriers, and lipid elongation can improve protease resistance, enhance the tendency to fold, and increase the overall bioavailability [4,5]. Moreover, peptide synthetic and conjugation techniques give us a tool to combine different epitope peptides in one compound that can reflect the antigen diversity of a pathogen [6].

In this study, epitope peptides derived from immunodominant proteins of *Mycobacterium tuberculosis* (*Mtb*) were evaluated. After infection, active *Mtb* bacterium can remain in the host phagocytes and renders itself to a phenotypically resistant form [7]. In this latent phase, the pathogen changes its gene expression and consequently the whole antigen repertoire. To enhance the efficacy of a vaccine against tuberculosis (TB), it is essential to combine T-cell epitopes derived from proteins that are expressed by active and latent forms of the causative pathogen [8]. In order to produce a multivalent vaccine conjugate, the following proteins were considered in this study:

- Ag85B (325 aa, encoded by the *Rv1886c* gene) is an early secreted antigen that is associated with active bacterial replication [9,10]. In the *C*-terminal region, CD8+ T-cell epitope peptide 239-KLVANNTRL-247 (**A**) was reported to lyse BCG-infected human macrophages and Ag85B-pulsed CD8 + T-cells [11,12,13].
- Isoniazid-inducible protein, IniB (479 aa, encoded by the *Rv0341* gene), is strongly upregulated in response to a broad range of inhibitors of cell wall biosynthesis, including isoniazid, a first-line antitubercular drug [14,15]. In the 33–45 region, an MHC I-associated 33-GLIDIAPHQISSV-45 peptide (**I**) was identified that can induce the generation of peptide-specific cytotoxic T-cells and lysis of *Mtb*-infected dendritic cells [16].
- PPE68 protein (368 aa, encoded by the *Rv3873* gene) is one of the major antigenic protein associated with dormancy and assists the pathogen in surviving within the host phagocytes [17,18]. Highly sensitive PPE68-specific T-cell epitope peptides within the 124–156 region were described recently [17,19,20], of which the 127-FFGINTIPIA-136 peptide (**P**) was identified as an HLA-DR promiscuous core sequence [21].

Empty MHC molecules on the surface of professional antigen-presenting cells are able to collect peptide antigens from the extracellular milieu for presentation to T cells. Nevertheless, larger peptides and conjugates that have entered the endocytic pathway of antigen presentation provoke a more efficient immune response. This observation leads to the utilization of synthetic long peptides (SLPs) that have proven clinical efficacy for tumor therapy [22] and against numerous autoimmune [23] and infectious diseases [24]. However, the step-by-step synthesis of long peptides is challenging. Incomplete coupling reactions and/or deprotection and aggregation of the growing peptide chain often result in deletion sequences and overall inefficiencies in the production of SLPs. Therefore, fragment condensation and chemical ligation techniques are often applied. Besides, branched-chain conjugates are designed to ameliorate synthetic difficulties. Nevertheless, the spatial arrangement is an effective way of disrupting peptide aggregation. 

Another challenge is to design a conjugate with epitopes that induces efficient cell-mediated immune response and delivers the epitopes to antigen-presenting cells. The elongation of peptide-based conjugates with lipid moieties has been shown to give them function as both adjuvants and delivery vehicles [1,2,25]. Previous studies have illustrated a role for the improved immunogenicity of self-adjuvanting lipopeptide delivery systems that contain C16 lipid (palmitic acid) with the advantage of increased cellular uptake by dendritic cells and activation of Th1/Th2 cytokine response [26].

In this work, the role of the palmitoyl elongation of a branched-chain epitope conjugate was studied. As a core sequence, tuftsin peptide was applied. Tuftsin, a natural immunostimulatory tetrapeptide (TKPR), has been found to increase macrophage-mediated phagocytosis, splenocyte proliferation, and bactericidal activity [27,28,29]. During the past decade, a new group of peptide carriers was developed in our laboratory: peptide derivatives consisting of the canine sequence (TKPKG) that exhibits tuftsin-like properties [30]. 

## 2. Materials and Methods

### 2.1. Materials

For peptide synthesis, amino acid derivatives were obtained from Iris Biotech (Marktredwitz, Germany). Reagents such as *N*,*N*’-diisopropylcarbodiimide (DIC), triisopropylsilane (TIS), 1-hydroxybenzotriazole (HOBt), 1,8-diazabicyclo[5.4.0]undec-7-ene (DBU), NH_4_OAc, palmitic acid, 6-maleimidohexanoic acid, acetic anhydride (Ac_2_O), *N*,*N*-diisopropylethylamine (DIEA), 2,2,2-trifluoroethanol (TFE), and 5(6)-carboxyfluorescein (*Cf*) were purchased from Sigma-Aldrich (Budapest, Hungary). Fmoc-Rink Amide MBHA resin was obtained from Merck. Trifluoroacetic acid (TFA), *N*,*N*-dimethylformamide (DMF), dichloromethane (DCM), 2-ethoxyethanol (Cellosolv), diethyl ether, and acetonitrile (AcN) were from VWR (Budapest, Hungary).

For the *in vitro* assays, RPMI-1640 medium, fetal calf serum (FCS), 5(6)-carboxyfluorescein diacetate *N*-succinimidyl ester (CFSE), concanavalin A (ConA), Mowiol^®^ 4–88, trypan blue, and trypsin were obtained from Sigma-Aldrich (Budapest, Hungary). Macrophage colony-stimulating factor (M-CSF) was from Shenandoah Biotechnology (Warwick, PA, USA). BBL Löwenstein–Jensen Medium was ordered from Becton Dickinson (Környe, Hungary), HPMI buffer (9 mM glucose, 10 mM NaHCO_3_, 119 mM NaCl, 9 mM HEPES, 5 mM KCl, 0.85 mM MgCl_2_, 0.053 mM CaCl_2_, 5 mM Na_2_HPO_4_ × 2H_2_O, pH = 7.4) was prepared in-house using components obtained from Sigma-Aldrich (Budapest, Hungary).

### 2.2. Synthetic Procedures

Peptides were produced on solid phase (Fmoc-Rink Amide MBHA resin, capacity = 0.67 mmol/g) in an automated peptide synthesizer (Syro-I, Biotage, Uppsala, Sweden) using a standard Fmoc/tBu strategy with DIC/HOBt coupling reagents. Fluorescently labelled derivatives were synthesized with the use of 5(6)-carboxyfluorescein with the DIC/HOBt coupling method. In the case of branched conjugates, one of the lysines in the sequence of the **AI** peptide was used in Fmoc-Lys(Dde)-OH protected form. The *N*-terminus of the **AI** peptide was acetylated with Ac_2_O:DMF:DIEA = 1:3:1.2 mixture (30 min, RT). In the case of the palmitoylated derivative (**pal-AI**), palmitic acid was introduced on the *N*-terminus of the **AI** peptide. After synthesis of the linear peptide chain (**AI** or **pal-AI**), the Dde protecting group was selectively removed with 2% N_2_H_2_/DMF solution (6 × 2 min) then 6-maleimidohexanoic acid was reacted with the free amino group in the presence of DIC/HOBt coupling reagents. Peptides were cleaved from the resin with TFA:H_2_O:TIS (9.5:2.5:2.5 *v/v*) mixture (2 h, RT). After filtration, compounds were precipitated in cold diethyl ether, centrifuged (4000 rpm, 5 min) and freeze-dried from water.

Maleimide derivative of **AI** or **pal-AI** peptide (50 mg) was dissolved in 10 mL 1:2 mixture of 2-ethoxyethanol (Cellosolv) and 0.1 M NH_4_OAc (pH = 5.2). Then, 1.1 equiv. of cysteine elongated epitope **P** was added to the solution and stirred for 12 hours. The solution was freeze-dried and purified by RP-HPLC.

RP-HPLC purification was performed on a ThermoFisher (Waltham, MA, USA) UltiMate 3000 Semiprep HPLC with a C-18 Phenomenex Jupiter column (250 × 10 mm) using gradient elution, consisting of 0.1% TFA in water (eluent A) and 0.1% TFA in acetonitrile:water = 80:20 (*v/v*) (eluent B). For palmitoylated derivatives, a C-4 Phenomenex Jupiter (250 × 10 mm) column was used.

Purified peptides were analyzed by RP-HPLC on analytical C-18 or C-4 columns using gradient elution with the above-mentioned eluents A and B (flow rate was 1 mL/min, UV detection at λ = 220 nm). The molecular mass of the peptides was determined using a Thermo Scientific (Waltham, MA, USA) Q Exactive™ Focus Hybrid Quadrupole-Orbitrap™ Mass Spectrometer. The peptide content of the final product was determined by amino acid analysis using a Sykam Amino Acid S433H analyzer equipped with an ion-exchange separation column. Prior to analysis, samples were hydrolyzed with 6 M HCl in sealed and evacuated tubes at 110 °C for 24 h. For post-column derivatization, the ninhydrin-method was used.

### 2.3. Electronic Circular Dichroism (ECD) Spectroscopy

ECD measurements were performed on a Jasco J-1500 dichrograph in a 0.1-cm quartz cell at room temperature. The solvent reference spectrum was used as baseline, which was automatically subtracted from the peptide ECD spectra. Band intensities were expressed in mean residue ellipticity ([Θ]_MR_, deg × cm^2^/dmol).

### 2.4. In Vitro Evaluation (Cellular Uptake, Cytotoxicity, and Hemolytic Activity Assays)

Internalization of the compounds was measured in the MonoMac6 (MM6) human monocytic cell line [31] and on murine bone marrow-derived macrophages (BMDMs). MonoMac6 cells were maintained as an adherent culture in RPMI-1640 supplemented with 10% heat-inactivated fetal calf serum (FCS) L-glutamine (2 mM) and gentamicin (35 μM) at 37 °C in a humidified atmosphere containing 5% CO_2_. For the generation of BMDMs, cells were isolated from tibiae and femurs of 6–12 week-old BALB/c mice, then cells were cultured in R-10 media containing 10 ng/mL M-CSF for 7 days. For the assay, cells were treated with peptides at 2.5 µM final concentration and were incubated for 2 hours. After centrifugation (1000 rpm, 5 min) and washing with serum-free RPMI medium, supernatant was removed and 100 μL 0.25% trypsin was added to the cells. After 5 min incubation, 0.8 mL 10% FCS/HPMI medium was added, then cells were washed and re-suspended in 0.3 mL HPMI medium. The intracellular fluorescence intensity of the cells was measured on a BD LSR II flow cytometer (BD Biosciences, San Jose, CA, USA) on channel FITC (emission at λ = 505 nm) and data were analyzed with FACSDiva 5.0 software (BD Biosciences, San Jose, CA, USA). External fluorescence quenching was performed by adding 10 µL of 0.04% trypan blue solution. All measurements were performed in triplicates, and the mean fluorescent intensity together with standard error of the mean (SEM) was graphically presented.

Cellular uptake and localization were visualized by confocal microscopy. MM6 cells were seeded (10^5^ cells/well) one day prior to treatment on cover glass containing 24-well plates (Sarstedt, Nümbrecht, Germany). Lysosomes were stained by LysoTracker^TM^ Deep Red (Invitrogen, Carlsbad, CA, USA) for 30 min, followed by incubation with *Cf*-labeled peptides for 60 min. Subsequently, nuclei were stained by Hoechst 33342 solution (Thermo Scientific, Waltham, MA, USA). After each step, cells were washed three times with serum-free medium. Cells were fixed by 4% paraformaldehyde for 15 min and mounted to microscopy slides with Mowiol^®^ 4–88. Imaging was performed on a Zeiss LSM 710 system with 40× oil immersion objective. Images were processed with ZEN lite software (Zeiss, Oberkochen, Germany).

The hemolytic activity of the compounds was measured on human erythrocyte suspension. Cytotoxicity was assayed on murine BMDM. These two experiments are detailed in the Supplementary Information.

### 2.5. Ethical Statement

Animal experiments were carried out in accordance with the guidelines of Directive 2010/63/EU and Hungarian laws, and were approved by the Hungarian Scientific Ethical Committee on Animal Experimentation under the following protocol number: PE/EA/2569-4/2016.

### 2.6. Mice and Immunization

For the immunogenicity study, 6–10-week-old female CB6F1 mice (a cross between female BALB/c and male C57BL/6) were caged and allowed ad libitum access to water and to a standard pellet diet. Vaccine candidates (50 μg peptide/100 μL PBS) were injected s.c. three times, two weeks apart. Six weeks after the last immunization, mice were euthanized, and spleens were removed under sterile conditions. Single splenocyte suspension was prepared by macerating the cells through a 70 µM cell strainer (Falcon, Corning, NY, USA). After the lysis of the red blood cells with ACK lysis buffer (2 mL), cells were washed and resuspended in 5 mL RPMI-1640 media.

### 2.7. T-Cell Proliferation and Cytokine Assays

For the proliferation study, the protocol of Quah et al. was used [32]. Briefly, splenocytes (1–2 × 10^7^/mL PBS) were incubated with 5 μM 5(6)-carboxyfluorescein diacetate *N*-succinimidyl ester (CFSE) for 3 min. Excess CFSE was removed by extensive washing with 20% FCS/RPMI. Then, CFSE labelled cells were plated (2 × 10^5^ cells/well) and cultured with the antigens (**P/A/I** mix, 2.5 µM) for 5 days. As positive control, concanavalin A (ConA) was used at 5 µg/mL concentration. The proliferation of CFSE-labelled cells was analyzed by flow cytometry, and the percentages of proliferated cells were presented as a diagram.

To measure the cytokine release, splenocytes were plated (2 × 10^5^ cells/well) and re-stimulated with the antigens (**P/A/I** mix, 2.5 µM). After 5 days of incubation, supernatants were assayed by LEGENDplex Mouse Th Cytokine Panel (BioLegend, San Diego, CA, USA) following the manufacturer’s instructions.

### 2.8. Vaccine Efficacy

All work with infectious *Mtb* was performed at the National Public Health Center, National Biosafety Laboratory in Hungary under biosafety level 3 (BSL-3) conditions. For infection experiments, mice were kept in ventilated cages in the BSL-3 facility. Mice were infected with a mid-logarithmic culture of *Mycobacterium tuberculosis* H_37_Rv (10^6^ CFU/mL in PBS, 200 μL, i.p.) three weeks after the final immunization. Weight gain and well-being of the animals were monitored daily. Seven weeks after, mice were euthanized, and their organs were removed. To determine the viable bacteria, a portion of lung and spleen were homogenized in a tissue homogenizer using ceramic beads (MagNA Lyser Green Beads, Roche, Basel, Switzerland) in Bouillon broth (2 times 60 s, 7000 Hz), then 100 μL of the supernatant was plated onto BBL Löwenstein–Jensen Medium (Becton Dickinson, (Környe, Hungary). Colony forming units (CFU) were counted after 4 weeks of incubation. For histopathological analysis, the lung, spleen, liver, and kidney of each animals were removed and fixed in 8% neutral buffered formalin for 24 h at room temperature before removing from the high-containment facility. Tissue specimens were dehydrated in a series of ethanol and xylene baths and embedded in paraffin wax. Sections (3–4 μm) were stained with hematoxylin and eosin (HE). For in situ visualization of the acid-fast bacilli, the Ziehl–Neelsen (ZN) staining method was applied on similarly pre-treated sections. Slides were analyzed in an Olympus BX53 microscope (Tokyo, Japan), and photomicrographs were obtained with an Olympus SC100 high-resolution digital color camera with the use of the Olympus cellSens imaging software platform (Münster, Germany).

## 3. Results

**Scheme 1:**
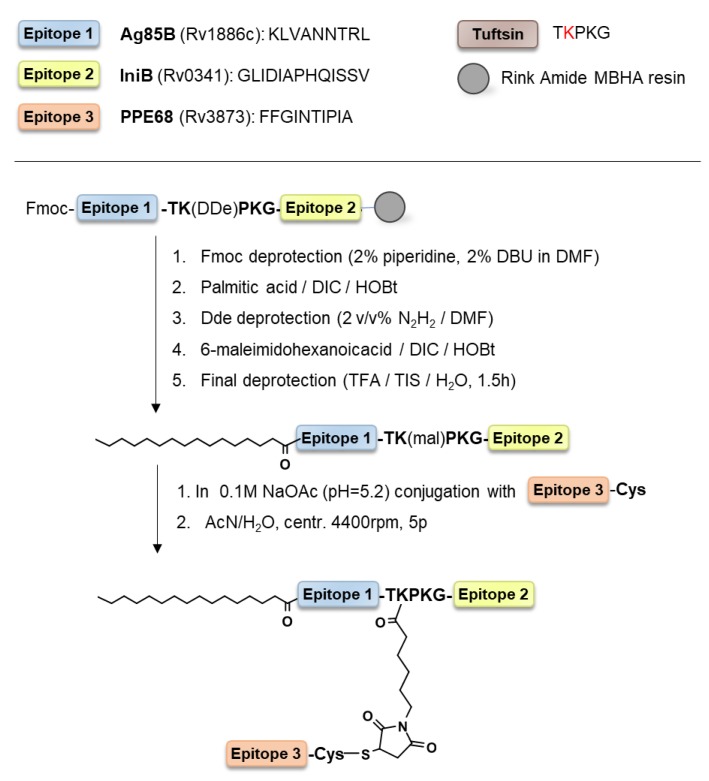
Outline of the synthesis of the **pal-A(P)I** conjugate.

The chimeric peptide KLVANNTRL-TKPKG-GLIDIAPHQISSV (**AI**) was designed to incorporate epitope peptide of Ag85B (**A**) and IniB (**I**) separated with a tuftsin sequence (TKPKG). The epitope of PPE68 (**P**) was attached to this peptide by the maleimide coupling strategy. The *N*-terminus of epitope ***P*** was acetylated (**A(P)I**) or lipidated with palmitic acid (**pal-A(P)I**) (Scheme 1).

### 3.1. Chemistry

**Scheme 2:**
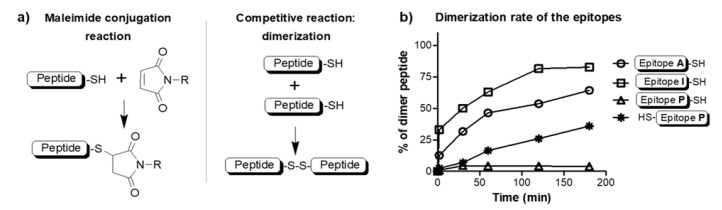
(**a**) Mechanism of maleimide coupling reaction and the competitive disulfide bond formation; and (**b**) the rate of dimerization of cysteine elongated epitope peptides.

The maleimide coupling strategy is a one-pot direct approach to attach a maleimide-peptide derivative to a cysteine elongated peptide. In the reaction mixture, the maleimide coupling reaction competes with the dimerization of the cysteine peptide (Scheme 2a). The dimerization of a cysteine-containing peptide strongly depends on the sequence and position of the cysteine residue. To distinguish which peptide arrangement would result in the best yield, all three epitopes were synthesized with cysteine, and the dimerization rate was measured. The slowest dimerization (disulfide bond formation) was measured for epitope **P** where the Cys residue was at the *C*-terminus of the peptide (FFGINTIPIA-C) (Scheme 2b). Therefore, this peptide was used further in the conjugation reaction.

Epitope **P**-Cys was allowed to react with the maleimide derivative of **AI**. The maleimide group was introduced on the solid phase after selectively removing the side chain protecting group of one of the lysines in the tuftsin core. The maleimide group remains intact during the final cleavage in the absence of nucleophiles such as thiol-based scavengers (i.e., 1,2-ethandithiol). It is also important to note that the Fmoc deprotection step should precede the coupling of maleimide because piperidine, which is used in the cleavage cocktail, reacts with maleimides. In the case of the palmitoylated derivative **pal-AI**, Cellosolv (2-ethoxyethanol) solvent was used in a mixture with 0.1 M NH_4_OAc (pH = 5.2). Cellosolv is an effective solvent for a wide array of apolar compounds, and is miscible with water. To solubilize a hydrophobic reactant, organic solvents such as *N*,*N*-dimethylformamide (DMF) or dimethyl sulfoxide (DMSO) are often used, but unlike DMF or DMSO, Cellosolv can be easily removed by lyophilization or rotary evaporation. After synthesis and purification, peptides and conjugates were characterized by high-resolution mass spectrometry, analytical HPLC chromatography, and amino acid analysis (Table 1).

Freeze-dried peptide products contain impurities of counter-ions and residual water. Net peptide content, measured by amino acid analysis, varied between 53.6% and 78.2% (Table 1). This is affected mainly by the amino acid composition and shows a strong correlation with the hydrophobicity of the peptides. Retention time on RP-HPLC chromatograms is a good indicator of the hydrophobicity of a peptide. For the peptides and conjugates, the greater retention time, the higher the measured peptide content.

### 3.2. Conformation Studies

**Figure 1:**
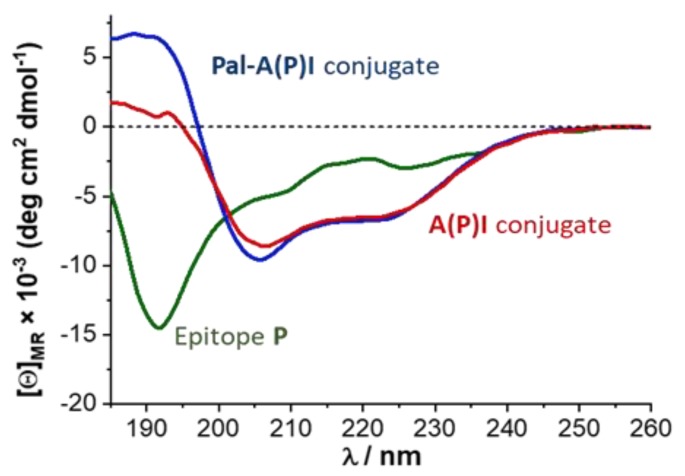
Electronic circular dichroism (ECD) spectra of the peptide and conjugates in trifluoroethanol (TFE).

Conformation of the peptides and conjugates was studied by electronic circular dichroism (ECD). The palmitoylated peptide conjugate **pal-A(P)I** is a hydrophobic molecule, characterized by very low water solubility. Thus, ECD measurements were performed in trifluoroethanol (TFE). This solvent promotes the formation of secondary structure. By the help of TFE, it is possible to investigate the propensity of a peptide to fold in a hydrophobic or lipophilic environment. Results show that the short, linear epitope peptides had highly dynamic conformation, even in TFE. In contrast, branched-chain conjugates had a much higher tendency to fold. The palmitoylated **pal-A(P)I** conjugate gave a more intense spectrum in the range of 185–210 nm, indicating a more partially folded state (Figure 1). These results reveal that branched conjugates were able to form an ordered structure (e.g., turn or helical) in a non-hydrophilic medium.

### 3.3. Immunological Evaluation

**Figure 2:**
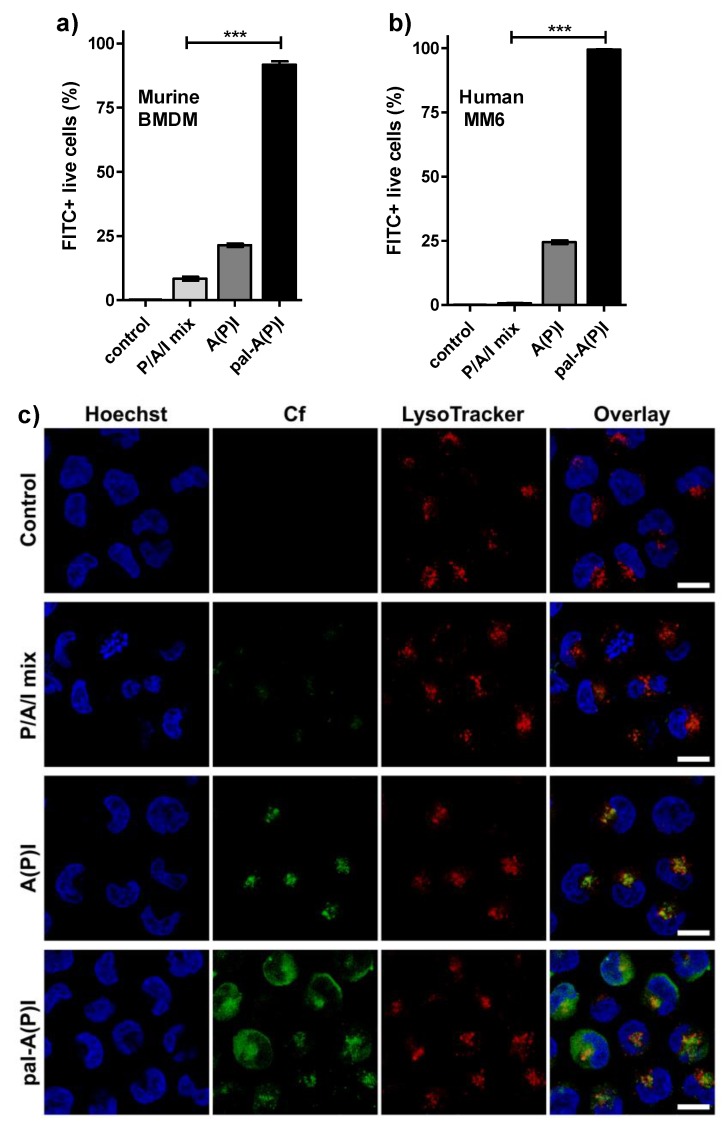
The internalization of *Cf*-labelled epitope peptides and conjugates (**P/A/I** mix, **A(P)I, pal-A(P)I**) was studied on (**a**) murine bone marrow-derived macrophages (BMDMs) and (**b**) MonoMac6 human monocytes (MM6) by using flow cytometry. (**c**) Localization of the compounds in MM6 cells were visualized by confocal laser scanning microscopy. Cells were incubated with *Cf-*labelled peptides (green), lysosomes were stained with LysoTracker Deep Red (red), nuclei were stained with Hoechst 33342 (blue). The scale bar represents 10 µm. (***: *p* < 0.001).

To study the role of conjugation and the role of palmitoylation, the following *Cf*-labelled compounds were compared: mixture of epitopes (named as **P/A/I** mix); branched conjugate **A(P)I**, and the palmitoylated conjugate **pal-A(P)I**. Internalization of peptides and conjugates to antigen-presenting cells was measured on two model cells, namely murine bone marrow-derived macrophages (BMDMs) and MonoMac6 human monocytes (MM6). The gating strategy of the flow cytometry measurements is shown in Figure S1. The cellular uptake of the **A(P)I** conjugate was higher than the uptake of the short epitope peptides (**P/A/I** mix). Furthermore, palmitoylation dramatically improved the internalization rate to human and to murine cells. The highest response was measured for **pal-A(P)I** (Figure 2a,b).

Localization of the compounds was visualized by confocal laser scanning microscopy in MM6 cells (Figure 2c). Cells were incubated with *Cf*-labelled peptides for 60 min; moreover, nuclei and lysosomes were also stained. The peptide concentrations for **P/A/I** mix, **A(P)I**, and **pal-A(P)I** were 5, 2.5, and 0.5 µM, respectively. As different compound concentrations and laser intensities were also used to visualize the compounds showing low or high internalization, the presented images give information about the intracellular localization and are not to be considered in a quantitative manner. The epitope peptide mixture (**P/A/I** mix) principally showed colocalization with lysosomes that was even more pronounced in case of **A(P)I**. **Pal-A(P)I** conjugate could be detected both in lysosomes and in the cytosol, which might indicate that this compound internalizes not only through the endo-lysosomal pathway, but also through direct cell penetration (Figure 2c).

**Figure 3:**
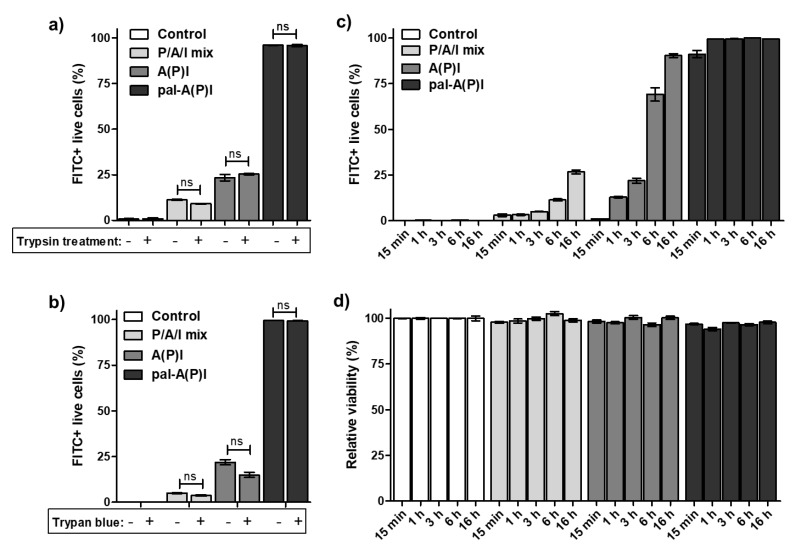
Detailed study on the cellular uptake by antigen presenting cells and the time course of internalization. Fluorescence intensity of compound treated cells was analyzed by flow cytometry (**a**) with or without trypsinization, and (**b**) in the presence or absence of trypan blue. No significant differences were observed after eliminating the fluorescent signal from adsorbed peptides or conjugates. (**c**) To study the time course of internalization, different incubation periods (15 min, 1 h, 3 h, 6 h, and 16 h) were applied, and the intracellular fluorescence intensity was measured. (**d**) The viability of compound-treated cells was compared to untreated control cells, and relative viabilities are presented. The viability of the control cells was always > 85% (results are represented as mean ± SEM, n = 3).

To accurately quantify the internalization of the peptides and to distinguish surface-bound peptides from internalized ones, external fluorescence was either quenched with trypan blue or membrane-absorbed peptides were removed by trypsin. Trypsinization is used to dissociate adherent cells from the plate in which they were cultured and, in the meantime, cell surface proteins and surface bound compounds are also cleaved from the cells. Trypan blue dye is non-permeant to live cells and is reported to quench the extracellular green fluorescence signal [33]. Cellular uptake of trypsinized cells showed the same rate as non-trypsinized cells (Figure 3a), and the percentage of FITC positive live cells was the same before and after the addition of trypan blue (Figure 3b). These results indicate that the peptides and conjugates were internalized rather than surface bound.

To further analyze the internalization of the compounds, the time course of the cellular uptake rate was measured by flow cytometry. As shown in Figure 3c, peptide-treated cells exhibited a time-dependent increase in the percentage of FITC-positive cells and in the mean fluorescence intensity. The internalization rate of **pal-A(P)I** conjugate was very fast. After 15 min incubation, the **pal-A(P)I** conjugate was inside almost all of the cells. Observations with **A(P)I** revealed that the internalization of this conjugate was significantly slower than the internalization of **pal-A(P)I**. Overnight treatment with **A(P)I** produced the same manner of intracellular fluorescence as 15 min treatment with **pal-A(P)I**. Results of flow cytometry and microscopy suggest that **A(P)I** and **pal-A(P)I** conjugates possess different internalization mechanisms and endocytic pathways.

The cytotoxicity of the compounds was measured on BMDM cells. Cells were incubated with the compounds for 3 h, then the viability was assayed by MTT test. Compounds **A**, **P**, **I**, **AI**, and **A(P)I** were not cytotoxic up to 100 µM concentration. The IC_50_ value of the palmitoylated conjugate **pal-A(P)I** was 76.0 ± 2.5 µM (Table S1). The hemolytic activity of the compounds was also evaluated using a human erythrocyte suspension. None of the tested compounds caused the hemolysis of red blood cells (HC_50_ > 100 µM) (Table S1). Based upon these results, peptides and conjugates were considered as safe and good candidates for *in vivo* functional assays.

**Figure 4:**
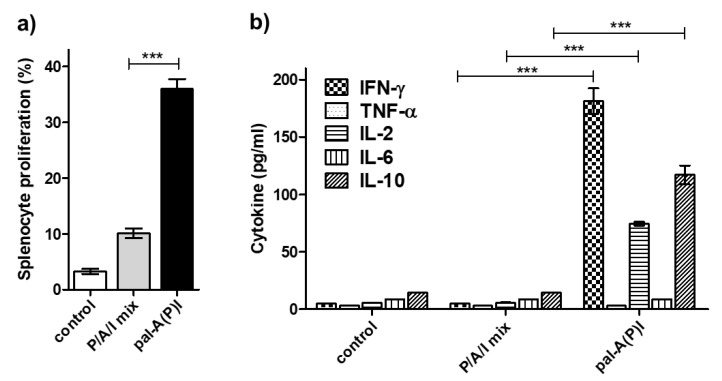
Immunogenicity of the compounds. (**a**) The provoked immune response was characterized in a T-cell proliferation assay, and (**b**) the cytokine response was measured by using a LEGENDplex bead-based immunoassay. Data represented as mean ± SEM are percent population of proliferated splenocytes (**a**) and pg/mL amount of cytokines (**b**) (n = 3 mice/group). (***: *p* < 0.001; TNF-α and IL-6: not significant).

To determine whether lipo-tuftsin conjugation could successfully improve the immunogenicity of T-cell epitope peptides, T-cell proliferation and cytokine release was measured using immunized CB6F1 mice. A group of animals was immunized s.c. with 50 μg of **pal-A(P)I** three times, two weeks apart. For comparison, a group was immunized with the mixture of the epitopes (**P/A/I** mix) using the same amount of peptides. Splenocytes from immunized animals were re-stimulated, and cell proliferation was assayed by CFSE staining method. Gating strategy and flow cytometry histograms are shown in Figure S2. Simultaneously, supernatants were tested for cytokine production using a LEGENDplex cytokine multiplex kit. Splenocytes from mice that had been vaccinated with **pal-A(P)I** conjugate showed a high proliferative response (Figure 4a), and re-stimulated cells produced around 180 pg/mL IFN-γ, 75 pg/mL IL-2, and 115 pg/mL IL-10 cytokines (Figure 4b). Immunization with free epitope peptides resulted in very low proliferative or cytokine response in all mice. These results indicate that the applied conjugation approach greatly enhanced the T-cell response to the epitopes.

### 3.4. Vaccine Efficacy

**Figure 5:**
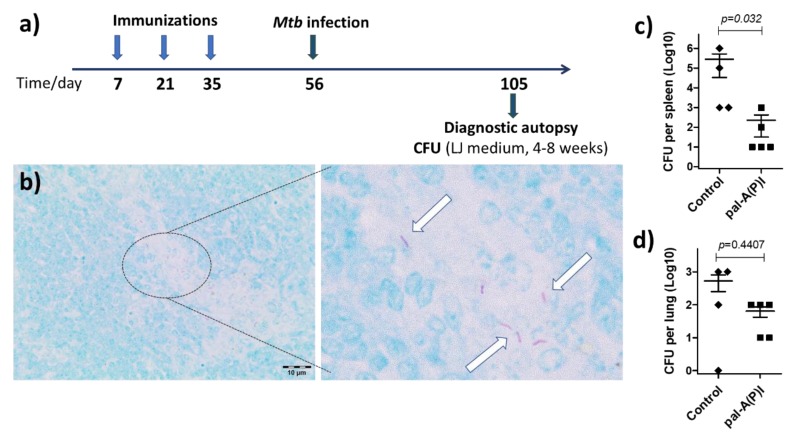
(**a**) Time schedule of the vaccine efficacy experiment and (**b**) experimental proof of *Mycobacterium tuberculosis* infection. In Ziehl–Neelsen-stained histologic sections prepared from the spleen, numerous acid-fast rod-shaped organisms were observed ((b), arrows point at *Mtb* cells). Magnification: 100× on the left panel, 600× on the right panel of (b). The vaccine efficacy of the **pal-A(P)I** conjugate was proved by enumerating a lower number of *Mycobacterium tuberculosis* (*Mtb*) bacteria from (**c**) spleen and (**d**) lung homogenates. Statistical significance was analyzed by Mann–Whitney U test (confidence intervals: 95%).

For the vaccine efficacy study, a group of five BALB/c mice were immunized with 50 μg **pal-A(P)I** s.c. three times, two weeks apart (Figure 5a). Then, mice were infected i.p. with 200 μL *Mycobacterium tuberculosis* H_37_Rv (10^6^ CFU/mL). Intraperitoneal administration was chosen for the bacteria as the i.p. route induces a course of slowly progressive disease [34]. Seven weeks after *Mtb* infection, mice were euthanized and autopsy was performed. Lung and spleen homogenates were cultured on Löwenstein–Jensen medium to determine the CFU (Figure 5c,d). In histologic sections prepared from the spleen of unvaccinated control animals, rod-shaped acid-fast bacteria were observed within small groups of epitheloid macrophages (Figure 5b). This indicates that the infection method used was successful in modelling experimental tuberculosis. When mice were immunized with the **pal-A(P)I** conjugate, lower numbers of bacteria were enumerated compared to the unvaccinated control group (Figure 5c,d). Tissue sections of **pal-A(P)I**-vaccinated animals were free of visible tuberculosis-related granulomatous lesions. With this experiment we have demonstrated the utility of this branched-chain conjugation technique to improve the immunogenicity and protective efficacy of T-cell epitope peptides.

## 4. Discussion

The aim of this study was to provide a simple synthetic route to improve the immunogenicity and vaccine efficacy of T-cell epitope peptides. Synthetic long peptides (SLPs) have proven clinical efficacy, but the production of long linear peptides is still challenging in most cases. Chemical design strategies to avoid synthetic difficulties such as the aggregation of the growing peptide utilize branched-chain conjugation techniques. Among procedures to make complex structures and multicomponent systems (i.e., azide–alkyne cycloaddition, lysine branching strategy, native chemical ligation, thioether, oxime, hydrazone or disulfide bond formation, etc.), one of the most convenient reactions is maleimide coupling. This type of conjugation is the transfer of a thiol group and a maleimide group to a thioether linkage. In most cases, maleimide moieties react with thiols very quickly, in an almost quantitative manner. The thioether bond is more stable than disulfide or hydrazone bonds, and therefore its application in bioconjugates is more reliable. Moreover, the maleimide group can be introduced as an inexpensive heterobifunctional linker with different lengths of spacer between the carboxyl and the maleimide groups. In this study, 6-maleimidohexanoic acid was used.

The conjugation reaction competed with the dimerization of the thiol-group-containing peptide. The slowest dimerization was measured for the peptide where the cysteine residue was at the *C*-terminus of epitope **P** (FFGINTIPIAC) (Scheme 2b). This observation was in line with previous studies that showed lower dimerization rates for *C*-terminal Cys compared to *N*-terminal cysteine-containing peptides [35]. In the design of conjugate, the peptide with the slowest dimerization was chosen to bear the thiol moiety, and the linear chain of the other two epitopes was decorated with the maleimide group.

The most important issue in vaccine development is to balance safety with efficacy. The use of minimal epitope sequences which can trigger the desired immune response is a safe approach, but the low immunogenicity needs to be addressed. Recent trends in synthetic vaccine research focus on the use of lipidic adjuvants that can be recognized by Toll-like receptors (TLRs), especially TLR2 and TLR4 [36]. Palmitic acid incorporation strongly improved the immunogenicity of the construct: both the T-cell proliferation and the cytokine production increased significantly compared to unconjugated epitope peptides. The T-cell response to **pal-A(P)I** conjugate was characterized by a Th1/Th2 cytokine pattern with prominent expression of IFN-γ, IL-2, and IL-10 (Figure 4b). Moreover, the **pal-A(P)I** conjugate showed a relevant vaccine efficacy in a slow-progression tuberculosis model experiment (Figure 5).

In summary, our study provides a convenient synthetic route to improve the immunogenicity of short, linear T-cell epitope peptides. Furthermore, a structure–activity relationship was observed—namely, the more folded structure showed higher cellular uptake. We assume that the enhanced immunogenicity is the consequence of the improved internalization to antigen-presenting cells. Finally, these results highlight the importance of the appropriate formulation of epitope peptides which allow the development of epitope-based vaccine candidates.

## Figures and Tables

**Table 1:** Analytical characteristics of the peptides and peptide conjugates.

Compound	Sequence	M_mo_ Calc.	M_mo_ Meas.^1^	Rt (min) ^2^	Peptide Content (%) ^3^
**P/A/I mix**	KLVANNTRL	1026.6298	1026.6295	8.4	53.6
	GLIDIAPHQISSV	1347.7510	1347.7508	10.7	58.2
	FFGINTIPIA	1090.6175	1090.6174	12.7	65.1
**AI**	KLVANNTRL-TKPKG-GLIDIAPHQISSV	2868.6661	2868.6679	10.2	58.5
**A(P)I**	KLVANNTRL-TKmal(FFGINTIPIAC)PKG-GLIDIAPHQISSV	4297.3535	4297.3649	13.0	64.5
**pal-A(P)I**	palmitoyl-KLVANNTRL-TKmal(FFGINTIPIAC)PKG-GLIDIAPHQISSV	4493.5726	4493.5757	24.1*	78.2
***Cf*-P/A/I mix**	*Cf-*KLVANNTRL	1384.6775	1384.6778	11.3	nd
	*Cf*-GLIDIAPHQISSV	1705.7988	1705.7986	13.3	nd
	*Cf*-FFGINTIPIA	1448.6653	1448.6654	14.8	nd
**A(*Cf-*P)I**	KLVANNTRL-TKmal(*Cf*-FFGINTIPIAC)PKG-GLIDIAPHQISSV	4655.4012	4655.4147	15.5	65.2
**pal-A(*Cf*-P)I**	palmitoyl-KLVANNTRL-TKmal(*Cf-*FFGINTIPIAC)PKG-GLIDIAPHQISSV	4851.6203	4851.6357	25.9 *	81.5

^1^ Exact M_mo_ (monoisotopic molecular mass) measured on a Thermo Scientific Q Exactive™ Focus Hybrid Quadrupole-Orbitrap™ Mass Spectrometer. ^2^ Retention time on an Agilent Eclipse XDB C8, 4.6 × 150 mm column, gradient: 5% B, 2 min; 5%–100% B, 20 min. * Phenomenex Jupiter C4, 4.6 × 250 mm column, gradient: 25% B, 5 min; 5%–100% B, 25 min. ^3^ Determined by amino acid analysis of the lyophilized final products. (nd: not determined). *Cf* is for 5(6)-carboxyfluorescein, the *C*-terminus of the peptides are in amidated form.

## Supplementary Materials

The following are available online at https://www.mdpi.com/2076-393X/7/3/101/s1, Table S1: Methods and results of the hemolytic and cytotoxicity assays, Figure S1: gating strategy applied in the cellular uptake measurement;, Figure S2: flow cytometry histograms of the splenocyte proliferation assay.
