# Supplementary material for: A Convenient Synthetic Method to Improve Immunogenicity of Mycobacterium tuberculosis Related T-Cell Epitope Peptides
Source: Vaccines (Basel). 2019 Aug 27;7(3):101. doi: 10.3390/vaccines7030101 (PMC6789589; doi:10.3390/vaccines7030101)
Supplement: Supplementary file 1 [file vaccines-07-00101-s001.pdf]

Supporting information for the paper entitled

A convenient synthetic method to improve immunogenicity of *Mycobacterium tuberculosis* related T-cell epitope peptides

Kata Horváti [1,6,*], Bernadett Pályi [2], Judit Henczkó [2], Gyula Balka [3], Eleonóra Szabó [4], Viktor Farkas [5], Beáta Biri-Kovács [1,6], Bálint Szeder [7] and Kinga Fodor [8]

[1] MTA-ELTE Research Group of Peptide Chemistry, Eötvös Loránd University, Hungarian Academy of Sciences, Budapest 1117, Hungary
[2] National Biosafety Laboratory, National Public Health Center, Budapest 1097, Hungary
[3] Department of Pathology, University of Veterinary Medicine, Budapest 1078, Hungary
[4] Laboratory of Bacteriology, Korányi National Institute for Tuberculosis and Respiratory Medicine, Budapest 1122, Hungary
[5] MTA-ELTE Protein Modelling Research Group, Eötvös Loránd University, Hungarian Academy of Sciences, Budapest 1117, Hungary
[6] Institute of Chemistry, Eötvös Loránd University, Budapest 1117, Hungary
[7] Research Centre for Natural Sciences, Hungarian Academy of Sciences, Budapest 1117, Hungary
[8] Department of Laboratory Animal and Animal Protection, University of Veterinary Medicine, Budapest 1078, Hungary
* Correspondence: khorvati@elte.hu

List of contents:

- Methods of the hemolytic assay and the cytotoxicity assay.
- Table S1. Results of the hemolytic and the cytotoxicity assays.
- Figure S1: Gating strategy of the cellular uptake measurement.
- Figure S2: Flow cytometry histograms of the splenocyte proliferation assay.

Hemolytic Activity Assay

The hemolytic activity of the compounds was assayed as described previously [1,2]. Briefly, peripheral blood from healthy volunteers was collected in vacuum tubes containing heparin (Li-heparin LH, VenoSafe) as anticoagulant. Tubes were centrifuged (1000 *g*, 5 min) and the pellet was washed three times with RPMI-1640 (culturing medium without phenol red). RPMI medium was added to the pellet to yield a final 2% (*v*/*v*) RBC suspension. DMSO stock solutions of the compounds were diluted with medium and three-fold serial dilution series were prepared (final concentration: 0.05–100 μM, DMSO concentration: 0.5%). RBC suspension (100 μL/well) were placed into a 96-well U-bottom cell culture plate and mixed with 100 μL peptide solution. The plates were incubated for 2 h at 37 °C. After centrifugation (1000 *g*, 5 min), 100 μL of the supernatant was transferred to a flat-bottom microtiter plate and absorbance was measured at 414 nm using an ELISA plate reader (iEMS Reader, Labsystems). The percentage hemolysis was compared to a hemolytic control peptide [2] (CM15, 200 μM) treated RBC and the concentration of peptide at which 50% hemolysis occurred (HC_{50} value) was determined. Compounds were measured in triplicates.

Cytotoxicity assay

The cytotoxic effect of the compounds was measured on murine BMDMs. Prior to treatment, cells were cultured for 24 h in serum-free RPMI medium (100,000 cells, 100 μL/well, flat-bottom 96-well culture plate). DMSO stock solutions of the compounds were diluted with medium, and two-fold serial dilution series were prepared (final concentration: 1.5–100 μM, DMSO concentration: 0.5%). Cells were treated with the compounds for 3 h, then cell viability was tested using MTT assay [3–5]. Briefly, 45 μL MTT solution was added to each well (2 mg/mL, solved in serum-free medium). Following a 5 h incubation, plates were centrifuged at 2000 rpm for 5 minutes, and the supernatant was carefully aspirated with a G30 needle. The precipitated purple crystals were dissolved in 100 μL DMSO, and after 10 minutes agitation, the absorbance was determined at λ = 540 nm and 620 nm using an ELISA plate reader (iEMS Reader, Labsystems). Cytotoxicity, expressed in percentage as the function of compound concentration, was graphically presented, and IC_{50} values were determined. Data are mean ± sd of four parallel measurements.

Table S1. Results of the hemolytic and cytotoxic assays.

**Compound**	**Hemolytic activity [1]**<br>**HC_{50} ± sd**	**Cytotoxicity [2]**<br>**IC_{50} ± sd**
**P/A/I** mix	>100 μM	>100 μM
**A(P)I**	>100 μM	>100 μM
**pal-A(P)I**	>100 μM	76.0 ± 2.5 μM

[1] Determined on 2% (*v*/*v*) human erythrocyte suspension.
[2] MTT assay on murine BMDM cells.

Internalization study

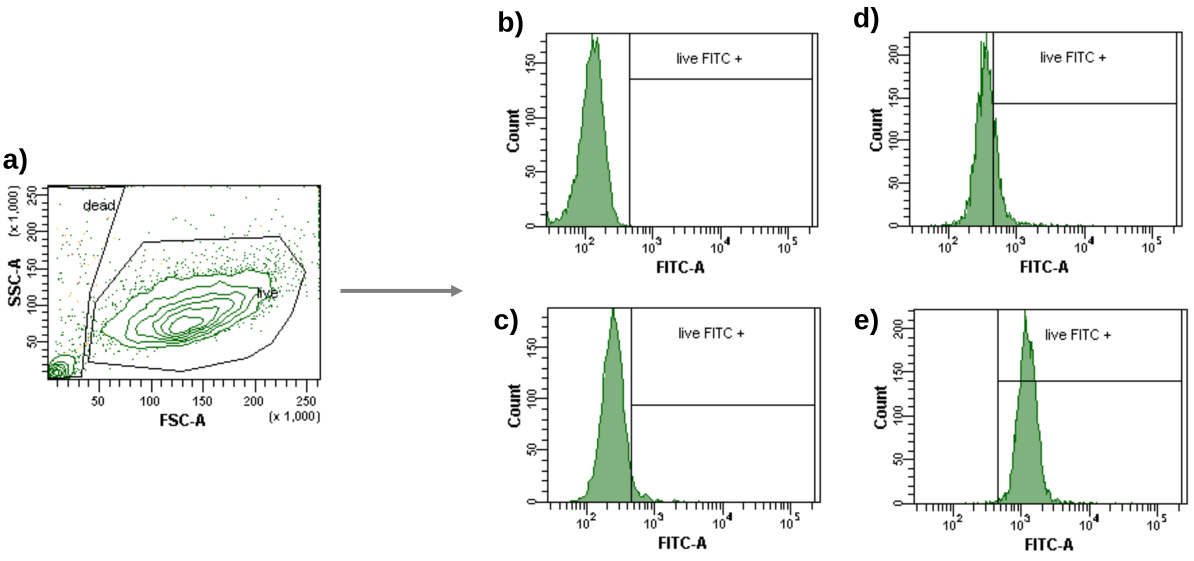

Figure S1. Gating strategy applied in the cellular uptake measurement. Human MM6 monocytes were treated with the compounds, then cells were analyzed by flow cytometry. To quantify viable cells, propidium iodide (PI) was added to a tube, and PI negative population was gated as live cells (**a**). As negative control, RPMI medium was used (**b**) and the FITC-negative population was determined on this panel. On the histograms, percentage of FITC-positive cells were determined ((**c**) **P/A/I** mix (4.9%), (**d**) **A(P)I** (21.5%), (**e**) **pal-A(P)I** (99.5%)).

Splenocyte proliferation

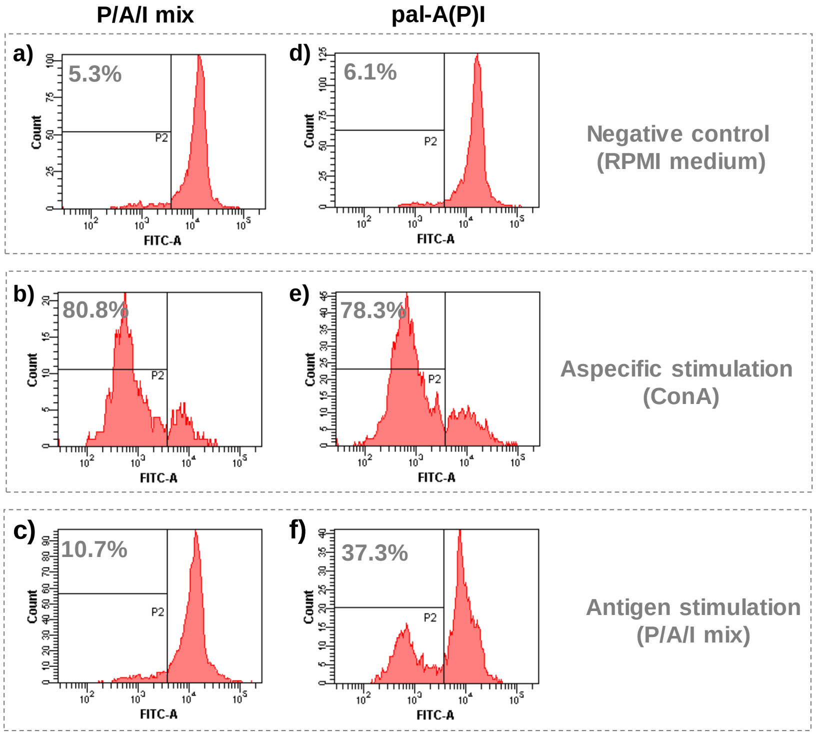

Figure S2. Splenocyte proliferation assayed by CFSE staining method. Mice were immunized three times with either **P/A/I** mix (**a**,**b**,**c**) or with **pal-A(P)I** conjugate (**d**,**e**,**f**), at an interval of 2 weeks. Six weeks after the last immunization, single cell suspensions were prepared from the spleens and cells were re-stimulated with the antigens (**c**,**f**) for 5 days. As negative control, medium was used (**a**,**d**); as positive control, concanavalin A (ConA) was applied (**b**,**e**). On the flow cytometry histograms, proliferated cells (P2 population) were gated and the percentage compared to all cells was calculated.

REFERENCES

[1] E. J. Helmerhorst, I. M. Reijnders, W. van't Hof, E. C. I. Veerman and A. V. N. Amerongen, *Febs Lett*, **1999**, 449, 105-110.

[2] K. Horváti, B. Bacsa, T. Mlinkó, N. Szabó, F. Hudecz, F. Zsila and S. Bősze, *Amino Acids*, **2017**, 49, 1053-1067.

[3] J. C. Stockert, A. Blazquez-Castro, M. Canete, R. W. Horobin and A. Villanueva, *Acta Histochem*, **2012**, 114, 785-796.

[4] H. Z. Wang, C. H. Chang, C. P. Lin and M. C. Tsai, *J Ocul Pharmacol Th*, **1996**, 12, 35-43.

[5] G. Nikkhah, J. L. Darling and D. G. T. Thomas, *Anticancer Res*, **1987**, 7, 902-903.
